# Supplementary material for: Spectroscopy Study of Albumin Interaction with Negatively Charged Liposome Membranes: Mutual Structural Effects of the Protein and the Bilayers
Source: Membranes (Basel). 2022 Oct 23;12(11):1031. doi: 10.3390/membranes12111031 (PMC9696317; doi:10.3390/membranes12111031)
Supplement: Supplementary file 1 [file membranes-12-01031-s001.zip › membranes-1973851-supplementary.pdf]

Supplementary material

Spectroscopy Study of Albumin Interaction with Negatively Charged Liposome Membranes: Mutual Structural Effects of the Protein and the Bilayers

Daria Tretiakova [1,*], Maria Kobanenko [1], Irina Le-Deygen [2], Ivan Boldyrev [1], Elena Kudryashova [2], Natalia Onishchenko [1,†] and Elena Vodovozova [1]

1 Shemyakin–Ovchinnikov Institute of Bioorganic Chemistry, Russian Academy of Sciences, ul. Miklukho-Maklaya 16/10, 117997 Moscow, Russia
2 Department of Chemistry, Lomonosov Moscow State University, Leninskie Gory 1/3, 119991 Moscow, Russia
* Correspondence: daria@lipids.ibch.ru; Tel.: +7-495-330-6610
† Current Address is Center for Soft and Living Matter, Institute for Basic Science, UNIST-gil 50, Building 103, Ulsan 44919, Korea.

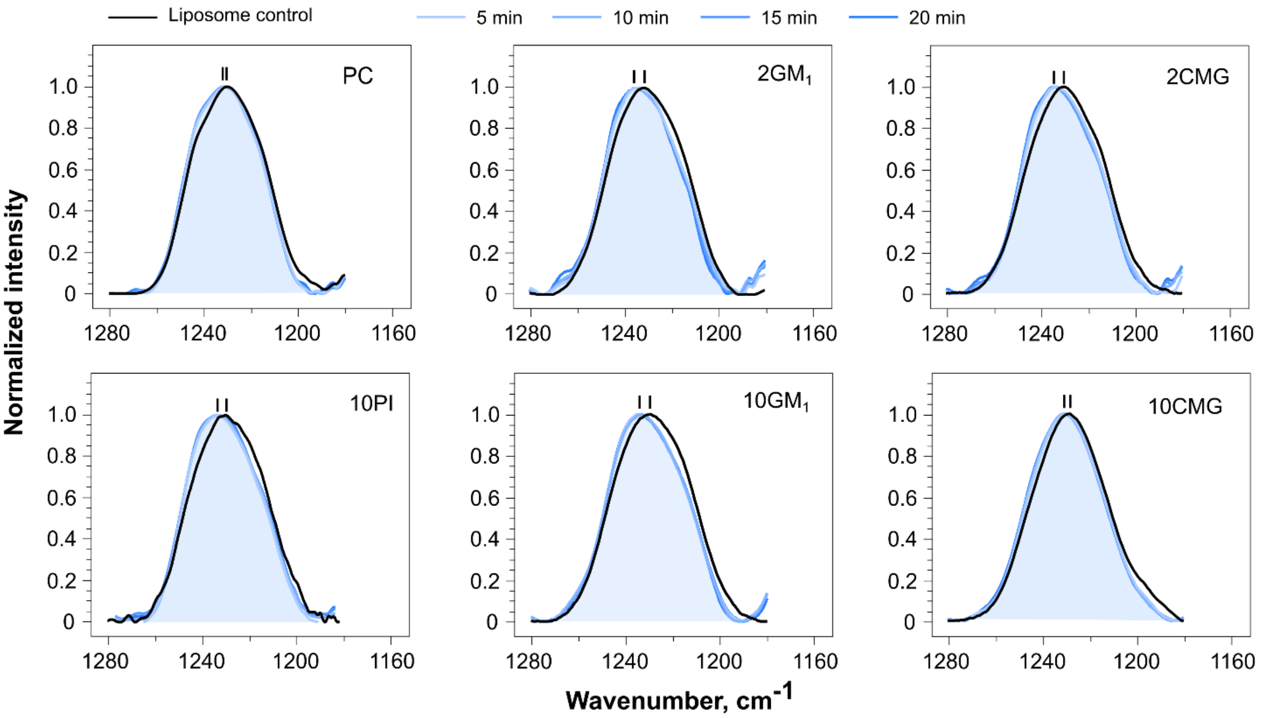

Figure S1. Normalized PO_2^- asymmetric stretching vibrations for liposomes alone (black) and upon their incubation with BSA (shades of blue). FTIR spectra were recorded in PBS at 37 °C, lipid concentration 12 mM, albumin concentration 6 mg/mL.

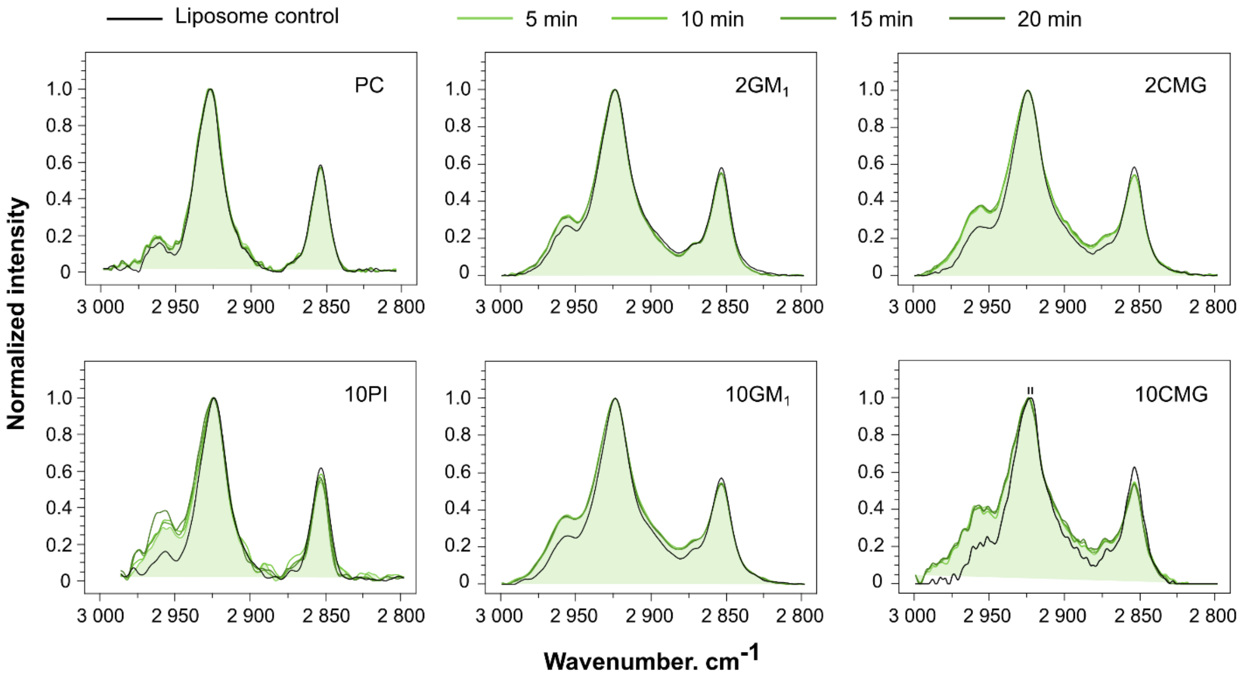

Figure S2. Normalized methylene asymmetric and symmetric vibrations for liposomes alone (black) and upon incubation with BSA (shades of green). FTIR spectra were recorded in PBS at 37 °C, lipid concentration 12 mM, albumin concentration 6 mg/mL.

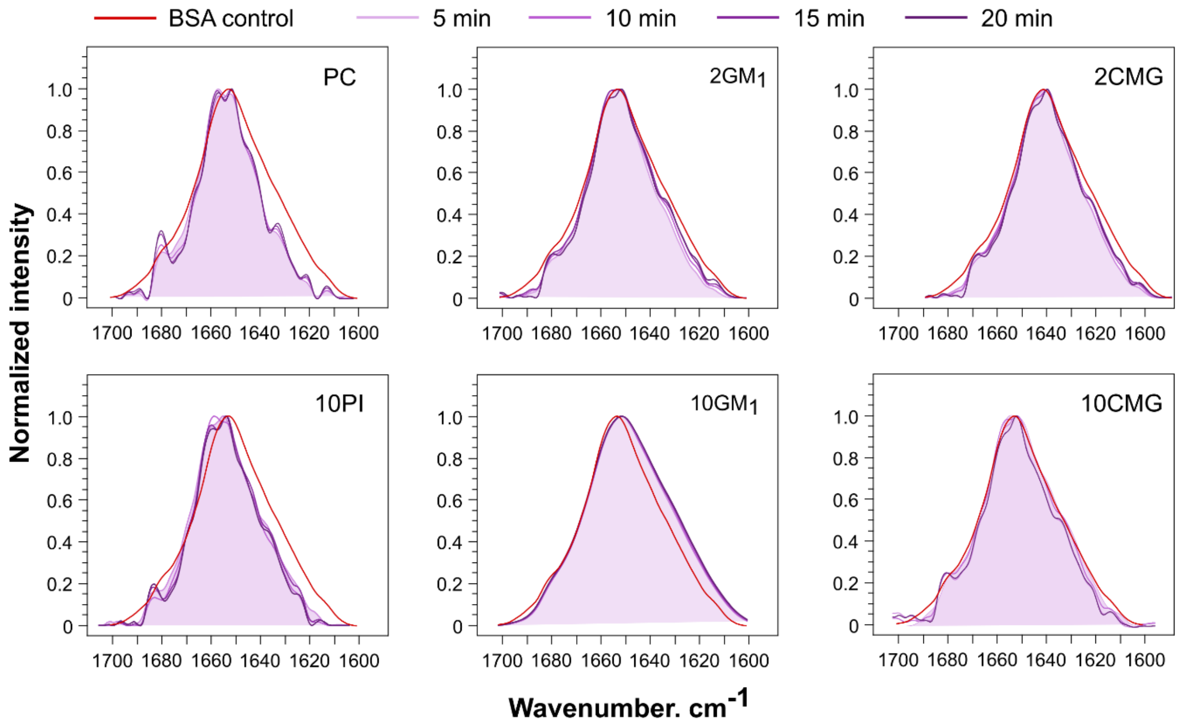

Figure S3. Normalized albumin Amide I peak alone (red) and upon incubation with liposomes (shades of purple). FTIR spectra were recorded in PBS at 37 °C, lipid concentration 12 mM, albumin concentration 6 mg/mL.
